# Perspectives of carers on medication management in dementia: lessons from collaboratively developing a research proposal

**DOI:** 10.1186/1756-0500-7-463

**Published:** 2014-07-21

**Authors:** Fiona Poland, Sarah Mapes, Hilary Pinnock, Cornelius Katona, Susanne Sorensen, Chris Fox, Ian D Maidment

**Affiliations:** 1 Social Research Methodology, School of Rehabilitation Services, University of East Anglia, Norwich Research Park, NR4 7TJ Norwich, UK; 2 NHS Anglia CSU (Commissioning Support Unit), Lakeside 400, Old Chapel Way, Broadland Business Park, NR7 0WG Norwich, UK; 3 Allergy and Respiratory Research Group, Centre for Population Health Sciences, University of Edinburgh. Doorway 3, Medical School, Teviot Place, EH8 9AG Edinburgh, UK; 4 Division of Psychiatry, University College London, Gower Street, WC1E 6BT London, UK; 5 Alzheimer's Society, Devon House, 58 St Katharine’s Way, E1W 1LB London, UK; 6 Psychiatry, Medical School, University of East Anglia, Norwich Research Park, NR4 7TJ Norwich, UK; 7 Pharmacy, Medicines and Devices in Ageing Cluster Lead, Aston Research Centre for Healthy Ageing (ARCHA), School of Life and Health Sciences, Aston University, B4 7ET Aston Triangle, UK

**Keywords:** Dementia, Service user engagement, Medicines management, Family carers

## Abstract

**Background:**

The need for carers to manage medication-related problems for people with dementia living in the community raises dilemmas, which can be identified by carers and people with dementia as key issues for developing carer-relevant research projects.

A research planning Public Patient Involvement (PPI) workshop using adapted focus group methodology was held at the Alzheimer’s Society’s national office, involving carers of people with dementia who were current members of the *Alzheimer’s Society Research Network* (ASRN) in dialogue with health professionals aimed to identify key issues in relation to medication management in dementia from the carer viewpoint. The group was facilitated by a specialist mental health pharmacist, using a topic guide developed systematically with carers, health professionals and researchers. Audio-recordings and field notes were made at the time and were transcribed and analysed thematically. The participants included nine carers in addition to academics, clinicians, and staff from DeNDRoN (Dementias and Neurodegenerative Diseases Research Network) and the Alzheimer’s Society.

**Findings:**

Significant themes, for carers, which emerged from the workshop were related to: (1) medication usage and administration practicalities, (2) communication barriers and facilitators, (3) bearing and sharing responsibility and (4) weighing up medication risks and benefits. These can form the basis for more in-depth qualitative research involving a broader, more diverse sample.

**Discussion:**

The supported discussion enabled carer voices and perspectives to be expressed and to be linked to the process of identifying problems in medications management as directly experienced by carers. This was used to inform an agenda for research proposals which would be meaningful for carers and people with dementia.

## Background

The prevalence of dementia is growing rapidly; in the UK 700,000 people currently live with dementia and this is predicted to double over the next 30 years [1,2]. As the condition progresses, people with dementia are increasingly less able to care for themselves making the role of family carers especially important for supporting them to continue to live in the community. People with dementia may receive many different medicines, both for the dementia and for other health problems [3]. However, medications can cause adverse health events [4]. Despite people with dementia being at particular risk of developing medication-related problems, little is known about the specific issues which relate to their medication management [5,6]. Building shared understandings is important for discovering what medication management issues may be seen as most relevant research topics for people with dementia and their carers living in the community.

Since the 1980s, there has been increasing international emphasis on involving the general public and service users in various aspects of healthcare [7-10]. Department of Health strategy documents have identified the involvement of service users and the public as crucial in all aspects of research [11]. Both the defining of topic and target population as well as appropriate and acceptable design approaches provide key opportunities for such involvement [12]. In dementia, as carers have a central role in medication management, they would need to be involved in designing any study. We therefore involved carers in identifying priorities for, and discussing the feasibility of a research project, focusing on the real-world experiences and needs of people with dementia and their carers.

The Alzheimer’s Society set up a volunteer group in 2000 to advise on its research funding programme, to ensure its greater relevance to people with dementia and their carers. Roles for volunteers include setting research priorities and scoring research applications for likely importance of outcome [13]. By 2011, the network had become central to Alzheimer’s Society’s research management, renamed *Alzheimer’s Society Research Network* (*ASRN* used for consistency in this paper) with 180 active members. Membership is restricted to people with dementia, carers and former carers for people with dementia. To promote the development of higher quality research projects in areas prioritised by the network, Alzheimer’s Society collaborated with the National Institute for Health Research (NIHR) Dementias and NeuroDegenerative Diseases Research Network (DeNDRoN) to organise an intensive workshop using modified focus group methods. Focus groups are frequently used in exploratory research, when little is known about the topic [14] and the group format may also facilitate the discussion of difficult and challenging experiences [15].

The developmental workshop on Medication Management in Dementia described here was initially proposed at a DeNDRoN Dementia Clinical Studies Group meeting. The lay members clearly articulated the particular importance of examining practice and communications around managing medication for the person with dementia including the practical problems involved. The workshop aimed to encourage a direct pharmacist-researcher-carer consideration of the dilemmas represented by carer members’ attempts to deal with medication issues, resonating with previous pharmacist investigations on the complexities raised [16]. The paper aims primarily to describe the PPI process which was intended to inform and validate the development of a future research proposal which could be well-informed by carers' perspectives.

## Method

### Workshop aim

A research planning PPI workshop using an adapted focus group methodology was held. The aim of the workshop was to develop an understanding of issues for a potential research study through identifying the carer’s views gained from experiences of medication management in dementia. Carer priorities, perceived benefits and side-effects of medication, issues for adherence, prescribing and dispensing, medication reviews, communication with healthcare professionals and any relevant ethical issues were explored. Understandings developed were then used to inform the design of a research proposal to develop systems to improve medication management for people with dementia. Reported thematic outcomes from workshop discussions were treated as part of this development process, posing questions to be explored in future research, but not being claimed to be established research findings. As this was not in itself a research project, it is therefore not reported using Consolidated Criteria for Reporting Qualitative Research (COREC) standards.

### Setting up and running the workshop group

In February 2011 an extended, supported workshop (two sessions of two hours each) was conducted with carers or ex-carers of people with dementia from *ASRN* who had received training about research processes and methodologies. Potential participants, who were members of the ASRN with experience of managing medication, were identified in two ways. First, we purposively identified ASRN members, who might have something specific to contribute to the theme based on their particular circumstances and previous discussions with them. Second, the whole membership was informed about the planned group in the general monthly mailing and those interested in participating were invited to contact the Alzheimer’s Society Research Office. Any members who contacted the Research Office were informed about the aims and objectives of the group. Based on these discussions, potential participants, purposively selected on the basis of specific criteria namely experience of medication management in dementia either as a carer or a person with dementia, were invited to attend the group (see Table 1 for details of the purposive sample of ASRN members who attended).

**Table 1:** Characteristics of carer workshop participants

Number of carers from the ASRN	9
Number of female carers	8
Ethnicity	White British (n = 9)

The group was facilitated by the mental health pharmacist (IM) supported by the qualitative researcher (FP) and the GP (HP); the Head of Research at Alzheimer’s Society (SS), the DeNDRoN Patient Public Involvement Officer, two administrators from DeNDRoN and a community pharmacist (SM) were also present. A topic guide was developed with the health professionals, researchers and the carers to ensure the systemic inclusion of carer voices (see Appendix 1).

An outline of the proposed exploratory workshop and the overall aim was sent to the local NHS Research Ethics Committee. On reviewing the outline, the committee concluded that ethical approval was not required. The focus group discussion was audio-recorded by a DeNDRoN administrative team member, transcribed by an experienced transcriber and supplemented by observation field notes by FP and SM. Participants gave consent for the recording by completing the Alzheimers Society’s Audio Consent Form.

### Analysis

The approach to analysis was informed by the need to ensure that the detailed perspectives participants were bringing to this PPI process were systematically identified and that the PPI process itself could be fully appreciated. A combination of thematic and narrative analysis itself was therefore used.

Thematic analysis was used to derive meaning units and themes from the transcripts, to summarise key features of the data, contextualising these within the wider data set [17]. The transcripts were analysed by initial codes developed by SM and FP; SM and FP initially independently coded the transcripts and then jointly discussed how they should be used to further develop themes.

The thematic analysts met repeatedly to review and agree emergent themes and to check that all relevant themes had been identified. The overall themes were validated with a third member of the team (IM) before being circulated for further review and agreement to all authors. Any differences were discussed with reference to the whole dataset. An element of narrative analysis was also used to demonstrate how themes were developed through the group’s conversation, again locating individual contributions within the whole dataset. This meant indicating those points which were firmly endorsed by a majority of carers, noting that some voices were stronger over the day and closely attending to and comparing the contributions of the less vocal.

## Findings

Four key themes that reflected the issues raised at the DeNDRoN Dementia Clinical Studies Group meeting by carers and prompted the convening of the workshop, were identified:

● Medication use and administration practicalities

● Communication barriers and facilitators

● Bearing and sharing responsibility

● Weighing up medication risks and benefits

### 1. Medication Use and Administration practicalities

The group opened with a prepared account of one carer’s difficulties with medication for constipation, including a demonstration of how quickly the laxative Fybogel® sets in the glass; much too quickly to be ingested by a person with dementia. This brought a consistent response from carers corroborating and elaborating the account. A sustained focus was brought to bear on the many practical implications of dealing with the appropriate use of medication for something that might be considered as a ‘minor’ problem (constipation) but which could entail severe emotional and physical distress for both person with dementia and carer:

P3 (Carer) *I…never talked about (it)…. it developed to such a point when my mother was actually packed with faeces….and it didn’t matter what I gave her in terms of what they tell you in the fact sheets.*

She related her own shock, shared by her listeners, of being abruptly told by a visiting nurse to deal with the unresolved problems by manually evacuating her mother’s bowels. She felt this contradicted many aspects of their previously shared relationship. This highlighted a particular and repeated feature of carers’ accounts of practical issues in medications management: the marked emotional weight such issues and corresponding responses from health professionals often carried for them.

Other carers reinforced their sense of despair in trying to communicate medication-related dilemmas:

P7 (Carer): *I would say to them, say no if you can’t do this, but I am desperate.*

The sensed lack of practical support from health and social care teams in dealing with medications issues was often stark. One carer (P6) with lengthy experience herself in social care reported, that her careful instructions to paid carers were ignored leaving her “so bloody annoyed”.

Several carers in the group defined professionals as necessarily removed from the practical consequences they dealt with at close quarters, as P2 (Carer) observed:

Don’t forget that the clinician and pharmacist can have little or no understanding of the practicalities.

For the experienced professionals in the group such emotion linked discussions made it very apparent how distant they were from the practicalities and realities of carers and people they cared for. P15 (Pharmacist researcher) described himself as “shocked by seeing and hearing this” and, never having seen how Fybogel® was made up, said that it “really looks revolting”.

Thus an apparently trivial and widely-used medication, could be seen to raise a host of unforeseen practical issues when having to be negotiated by family carers of people with dementia whose experience and cognitive functioning led to very different needs to be considered.

Carers described how more visible signs and symptoms were easier to look out for and helpful in explaining to someone with dementia, whose cooperation they needed to gain, by saying:

P3 (Carer) *this is to make them (your ankles) less puffy so there’s something very visible that she (person with dementia) could see…..but for other medications it’s more difficult to explain why they need to take it.*

These discussions led to concerns about many other types of medication, including those relating to pain relief, hypertension, osteoporosis, diabetes and eye problems. Each could be seen to have particular requirements for how and who should administer these as P12 (Carer) related:

My mother was living alone when she developed the signs and symptoms……I started to be concerned about the prescriptions that she was getting for her eye drops because I knew they had to be administered properly, but who would know?

This raised the issue of whether the carer or the person with dementia controls use of medication in practice, and, relatedly, how the carer can decide whether the person with dementia can no longer safely administer medication. P2 (Carer) described subsequent challenges as “a nightmare” complicated by the unfamiliar terminology used by health professionals and ambiguity about responsibility for monitoring their use:

who monitors as required (PRN medication)…Sometimes you get an agency nurse sent into just to do the drug round or a relative who do you ask whether they think it’s PRN and that was one of the things that was caused a lot of my husband’s distress…….he actually got it everyday. That’s not PRN so there’s a lot of ambiguity.

The examples cited here show graphically how the medications management practicalities raised for carers were not specific to drugs for dementia. However, managing the medications for other pathologies was complicated by the need to take into account the changed experience and capabilities of the person with dementia, which can transform the medications-related work that may have fitted almost unnoticed into everyday routines of people when they were not living with dementia. Striking in these accounts, and readily recognisable by the health professionals here, was that such practicalities were not experienced as neutral for carers, often causing high levels of embarrassment in which their identities and expectations as family members were undermined. All of this created uncertainty and further questions about how they might communicate within the family and with health professionals to gain useful knowledge and regain a sense of control. Barriers and facilitators to such communication are explored in the following theme.

### 2. Communication barriers and facilitators

Changes in the nature and quality of communication with health professionals could be experienced as pivotal to carers’ ability to engage with medication issues initially appearing insuperable:

P3 (Carer) *After months I got it absolutely perfectly balanced with a lot of help from the District Nurse….she went way beyond what she should have done….I will be eternally grateful to her …*

Here, building a supportive relationship with a clinician was seen as an important facilitator to communication, as another carer’s (P8) later comments also illustrate:

the most important thing…..…was the fact that she had direct access to this District Nurse which made an absolute difference if you have an access point and you have good communication.

Complex issues of embarrassment about disclosure of their relative’s loss of dignity and/or of their own perceived lack of knowledge could entail powerful barriers to communication:

P5 (Alz Soc Researcher) *it’s the embarrassment factor that …we might actually need something for constipation so should there be prompts.*

P3 (carer) thought that men, who were carers, found it especially difficult to discuss personal care issues:

Often men cover up for their wives……

Again a sense of shame and betrayal was graphically demonstrated when carers spelt out what care problems they were dealing with; articulated by some carers as betraying their relation’s trust in them to preserve confidentiality:

P8 (Carer) *it was my husband; I felt almost unfaithful exposing him.*

However, confidentiality issues could also be caused by professional codes which focused on the person with dementia and prevented sharing of knowledge with carers, leaving carers feeling in the dark and unsupported:

P11 (Carer) *The GP was trying to keep private confidential information, but it was extremely frustrating for us wanting to get some support.*

Carer-health professional discussion became particularly animated when elaborating the need for and possibility of medications advice which carers could keep ready to hand, perhaps as a checklist, listing key information, in lay terms, on medications (their effects, side effects and usage instructions etc). All carer participants expressed active agreement with this as a means to be informed and communicated with about medications, as P2 (Carer) suggested:

Some kind of check list…our own simplified version of the BNF (British National Formulary; a textbook designed for clinicians with guidance on medicines management).

However, the issue of indicative labelling on medications packaging demonstrated how barriers to providing simple information or instructions might arise. Health professional participants pointed out that a drug might have more than one use:

P10 (Pharmacist) *quite often there are multiple uses for a single drug.*

So it would not always be immediately obvious what labelling would be appropriate. Neither was it necessarily clear when and how such information might need to be given as some professionals and lay members had divergent views on whether such information-giving would improve patient care:

P14 (GP) *We don’t know yet whether it works because I’m afraid there's no evidence.*

P8 (Carer) *The pharmacists are saying it would be helpful.*

P5 (AS staff) said that previous discussions had suggested the potential of a prompt list to encourage people to ask the questions that “perhaps wouldn’t come by themselves” and went on to ask how carers and professionals might use or accept such questions. Carers and professionals discussed at length where simplifying such information-seeking and information-giving, particularly for the most common side effects could ease decision-making.

P8 (Carer) *Please, a simple prompt list.*

Carers offered highly-specific suggestions about how such checklists might be used in nursing homes to assist medication management, for example stuck to the “drug trolley” and also to flag up when action might be needed:

P2 (Carer) *before you give the drugs if something occurs ask the GP or go back to the pharmacist …*

The impact of the quality of the communication with professionals on carers’ decision-making responsibilities is explored next.

### 3. Bearing and sharing responsibility

Another strong theme was the carers’ detailed consideration of medication management as bringing a heavy burden of responsibilities, which they felt might be eased through sharing it with appropriate health professionals. However, as seen in the first two themes, this could not be counted on.

The nature of that burden was closely linked to carers’ anxiety about whether they could care well enough:

P8 (Carer) *I really do wish you wouldn’t ask me how I’m coping because the word coping implies that if I’m not, it’s my fault.*

They also talked about their additional sense of failed responsibility if they could not manage medications by noticing and responding to changes in the health of the person they care for:

P2 (Carer) *it would be neglect and carelessness to carry on giving laxatives when they have diarrhoea.*

As well as the requirement to “read” the health care and medication needs of the person they care for, the carer also needed to translate experts’ advice:

P13 (Carer) *the carer….interprets the advice of the experts.*

As individuals’ dementia progressed, this increased such difficulties for carers in communicating with them to ascertain their needs, again risking “letting them down”:

P12 (Carer) *She had no communication except for the tearful eyes and I thought god (I have) really let you down.*

Such comments indicate how, especially in relation to medication, carers cannot be sure they are adequately informed to fulfil such responsibilities. They went on to consider how having clearer information could help them fulfil their responsibilities:

P2 (Carer) *One of the things also that us and paid staff need better to do is powers of observation, it’s not just a checklist, and notice if somebody’s got a rash.*

Carers also saw it as part of their role to help empower people with dementia in relation to medication including seeking ways to support the person they cared for retain control of medications:

P12 (Carer) *I think she felt a loss of independence when the dementia hit. But not only that but as soon as her own control over her own medication, that she was so used to, was taken out of her hands….so it’s giving the autonomy to the patient as far as possible.*

The monitored dose medication system, designed to support administration and usage of medication by carers, again attracted opposing views from carers, who found them useful and health care professionals who saw them as “systems abused by care homes” (P15, Pharmacist researcher) and “desperately frustrating” (P14, GP).

Where carers could trust the quality and commitment of their support team they were more likely to share such responsibilities with them. However, a striking observation, especially in this pharmacist-facilitated discussion about potentially-helpful professionals, was carers’ general lack of awareness of pharmacists despite recent efforts to raise their profile:

P10 (Pharmacist) *We have an "ask your pharmacist week" don’t we, but we’re obviously not promoting ourselves enough.*

Overall, this theme helped highlight how uncertainty and poor communication added to carers’ difficulty in sensing how to share their heavy burden of responsibility for difficult medications decisions.

### 4. Weighing up medication risks and benefits

As the discussion developed, carers expanded on the burden of responsibility they carried in having to make decisions about whether benefits of their relative taking medications outweighed the risks, experiencing guilt and self-remonstration when they later felt their decision had led to ill-health:

P3 (Carer) *I carried on giving my mother her diuretics actually she was dehydrated. Neither the doctor nor I thought and I thought afterwards what an idiot I should have made that connection myself.*

This instance exemplified carers' difficulties in weighing up risks with benefits in the absence of access to expert knowledge about medications.

Some aspects of the dementia care situation, as with challenging behaviour, make it harder to balance the benefits of medication with harms to reputation and moral sense as well as health:

P9 (Carer) *what happens if you’ve got someone who is a violent patient…and the family have refused to give permission for the person to have drugs.*

Several carers mentioned the potential stigma of being known to use anti-psychotics even when their experience suggested that they might be appropriate:

P8 (Carer) *My husband would have been humiliated….if he had recognised his aggression and his nastiness with people so anti-psychotics have got a place and…..the bad press that it gets makes it very difficult.*

In such ways, carers’ accounts underlined their acute awareness of needing to balance the harms (including shortening life) as well as benefits of medications:

P2 (Carer) *It’s length of life versus quality of life and that’s something that we as carers we’re very loathe to face*.

This may resonate with recently-emerging ideas about time to benefit, increasingly considered in end-of-life care, where a medication may be dispreferred if it takes longer to produce benefits than the predicted life expectancy, suggesting that dementia-specific evidence may to help ease carers’ concerns [18].

While the potential harms from drugs readily raised fears for carers, there was no corresponding sense in the experiences shared within this group that it was easier to decide on when it might be beneficial rather than harmful to stop drugs; although some carers were prepared to face the issue:

P11 (Carer) *We stopped giving my mother the calcium altogether because she’d had a bone scan and it had been reasonably ok.*

Several carers raised, but understandably did not want to face, the possibility that stopping medication might be a sign of potentially losing the person they cared for:

P8 (Carer) *And I don’t think anyone wants to face it really*

Furthermore, even where the quality of life issues appeared clear in the abstract, carers agreeing to withhold medication was not readily experienced as right to do:

P3 (Carer) *She (a friend’s wife) had chest infection after chest infection and in the end he talked with the doctors and they agreed if she had another one because her quality of life was so bad, they wouldn’t treat and he rang up after she died and said I don’t know whether I can live with the guilt of what I’ve done you know I’ve let her die.*

Carers used terms such as “drug cocktail” to connote random mixtures without individual benefit, or talked of the “chemical cosh” in damning terms. The administration of sleep medication was an especially challenging decision as promoting sleep for both the person they cared for and allowing the carer more rest, could be seen as administering unnecessary drugs only in their own interests. As P7 (Carer) recounted:

There’s also the question of whether the carer requests it or whether this is all unethical. Again it’s because the only time I had a drug for my mother was when she wasn’t sleeping and therefore I wasn’t sleeping so I said she’s going to have something to put her out at night.

This raises the challenge of directly counterposing the differing needs of the carer and of the person with dementia, and of who might advocate for the person with dementia in medications administration. Carers provided mutually reinforcing examples of the weight of conflict imposed on them:

P7 (Carer) *My father he wandered and if you don’t get any sleep at all you get ragged*

Carers were keen to suggest that the need for medication-based solutions for behavioural issues might lessen, if a supportive relationship with a clinician could be established. P11 (Carer) went on to underline that having more support to reduce their isolation (so “bearing and sharing responsibility”) might reduce the need to consider “drugging:”

I had nights I was dealing with her on my own to begin with and I was absolutely worn to a frazzle. If eventually I got the support…you can manage….you should be caring for these people not drugging them up.

### Reflections

This paper has set out an initially carer-led process and outcomes from facilitating carers of people with dementia to identify and prioritise issues for research relating to medications use [11,12]. The methods used drew on carer-professional-academic engagement in a dementia-specialist clinical study group meeting, planning a focus group of mainly carers, systematically supported to contribute through a facilitated extended workshop from which contributions were thematically analysed [17].

The workshop provided rich articulation of the process of developing research. The “thick description” available in detailed stories from carers’ personal experiences provided nuanced insights into how they identified challenging emotional, practical, ethical and conceptual issues, described in context, within mutually supportive dialogue. These allowed issues for study and design, to be defined and prioritised. Viewing them holistically helped critically re-appraise those complex health and social care partnerships within which carers needed communication and support to deal with medications management.

The focus group discussion made clear that managing medication in dementia is neither trivial nor emotionally neutral for carers and patients. Furthermore, since this workshop was conducted a recent narrative review identified the complexity of the role and the need for further research [19]. The depth and range of emotions expressed and the variety of specific dilemmas and decision-making confronted by carers, provided powerful, detailed reasons to carry out research here and pointed to areas of most relevance. The discussions highlighted the need to understand why health care professionals might not spend time listening to patients and carers so as to address their medication-related difficulties and concerns. As pharmacist participants noted, while they routinely talk to individuals daily, the workshop confronted them with effects of different ways of eliciting carers’ key concerns. This underlined the value of facilitated discussion for better recognising the day-to-day practical difficulties in medication management from carers’ perspectives, so as to facilitate more meaningful and constructive carer-health professional communication.

Emergent themes identified the need for clear recognition and sharing of understandings of and communication around medications, particularly as many medications which raise relatively few management problems in people without dementia, confronted dementia carers with a host of practical, emotional and ethical problems, whose resolution requires further research. There was a lack of awareness of potential support, particularly of services available from pharmacies.

This discussion within the workshop also enabled carers to suggest areas for developing interventions to be tested such as information checklists to ease administration and labelling to support carers’ decision making. Carers’ graphic descriptions of issues which challenged them in managing medication helped indicate features which would be important for carers to be recruited to any studies of interventions [12]. Within these discussions, the power of shared experience was seen to strengthen the case for identifying as important, specific details to be considered in developing interventions, perhaps balancing the formal weight given to more “objectively evidenced” issues [20].

Bringing health professionals into dialogue with carers in an extended workshop may have brought more perspectives to demonstrate the extent of difference in their experience, understanding and weighting of medications issues. However, this may also have brought some potential limitations, now examined.

### Strengths, limitations and implications for carer involvement

A strength of this examination of the PPI development process was that the workshop allowed lay carer participants space and time to discuss issues important to them, in ways and language comfortable for them, with peers and Alzheimers Society staff with whom they had built a long-term relationship. This also helped dementia researchers and health practitioners to engage in research-related dialogues with carers and to see how incorporating carers’ perspectives could enhance the quality and significance of research being developed.

The reported thematic outcomes have been treated as part of this development process, posing questions to be explored in future research, but not being claimed to be established research findings. A further strength is the rigour of analysis applied to the examination of carer views expressed in this consultative process, unusual for such exercises. Nonetheless, as this was not a research project in itself it is therefore not reported to COREC standards.

A relative limitation might be that the findings are based on one workshop with a purposive sample within the sampling pool of a single organization albeit with a national membership. As such they may have wider indicative resonance but not generalisability. A further limitation is that whilst via the Alzheimer’s Society network both carers and people with dementia were invited to participate only carers attended the event.

Limitations may also have followed from the event’s duration. As the day went on and more technical aspects of medications were discussed, far fewer carers seemed to have the energy or interest to engage with mostly health professional-initiated issues. Therefore, only the earlier parts of the workshop, with which carers were more fully engaged, were used in reporting thematic development. An extended workshop may require more responsive and reflexive attention to processes to sustain and empower lay participants’ continued engagement and ownership of discursive directions, particularly as the group environment, in addition to being empowering, may inhibit people from discussing certain issues [15]. Constantly reviewing the process with all participants and providing more preparation time and facilitator planning would strengthen carer empowerment, to maximise their potential contribution.

## Conclusions

Carers face many challenges in supporting relatives with dementia as symptoms progress, often alongside multi-morbidities. Family carers are often confronted with difficulties connected with medications management and lacking appropriate forms of support. Directly involving carers in specifying topics that matter to them, can greatly enhance the relevance and feasibility of research to develop effective interventions. This paper has highlighted ways of supporting carers to articulate their experiences in order to develop meaningful research topics and processes.

A focus group of carers from a “consumer” research network with facilitated shared discussion of issues already identified by them as important and with contributions from interested health professionals was seen to substantially contribute to proposals for researchable topics. While this allowed a more relaxed, lay-oriented discussion, it was also seen to place demands on facilitators to constantly review participants’ energies and interests as discussion progressed. Embedding the workshop membership and event within longer term processes and networks for supporting carers’ voices in developing research helped prepare carers to take part in this research development process on their own terms while building dementia researchers’ and health practitioners’ experience of engagement in dialogues to enhance research development.

The problems faced by carers were richly demonstrated and their key priorities for developing interventions were contextualised in their lives and working partnerships with health professionals. The emotional costs, the communications gaps and complex ethical decision-making burdens emerged as issues to be considered as research topics, and should also inform research approaches to be adopted for successfully recruiting and engaging carers and people with dementia in related research. The workshop highlighted the complexity of developing interventions to facilitate effective medication management while helping scope some priorities as feasible and desirable for research. The focus group was primarily set up to ensure that research in medication management in dementia can adequately recognise the importance of carers’ roles and responsibilities. The power of the carers’ contributions was amply demonstrated in the ground covered, which clearly indicated health professionals’ major knowledge and practice gaps and that assumptions that medication management practices in dementia care in the community were working without problems are false.

## Appendix 1

### Topic Guide for Workshop - Perspectives of carers on medication management in dementia

1. Medication in dementia. Overview

– What do you think is involved in managing medicines?

– [Probe: what sorts of things do people need to do? How easy are these to arrange? Do risks also have to be managed? What sorts of responsibilities are involved? Who takes responsibility?]

2. Is it important for you? If yes, why?

– [Consider] What is the potential impact on the person with dementia?

– What is the potential impact on the carer e.g. do you find medication management activities relatively easy, or stressful?

3. Brainstorming session – flip chart to consider the important aspects of medication management.

4. Practical aspects of medication management

– How do you encourage compliance/adherence?

– How do you decide whether the person in your care for is capable of self-administration, or whether you need to administer the medication?

– How do you decide whether medication, including as required, is needed?

– Are there any issues regarding communication with healthcare professionals including confidentiality issues?

– How easy is it to maintain a continuous supply of medication for the person you care for?

5. Need for carers to make clinical-type judgements

– How do you identify and manage side-effects in the person with dementia?

– How do you decide whether treatments for cognitive impairment and dementia associated challenging behaviour are working (particularly bearing in mind fluctuations over the course of the day)?

– Do you have a role in identifying any mistakes e.g. checking that the medication details are correct – particularly for people with dementia transferred between primary and secondary health services?

6. Potential interventions to improve medication management in the population

– What specific problems (or bits of bad practice) regarding medication management pertaining to dementia have you ever encountered?

– Can you think of any elements of good practice relevant to medication for dementia that could or should be implemented?

– What possible solutions could you think of to improve medication management in dementia?

## Competing interests

The authors declare that they have no competing interests.

## Authors’ contributions

IM, FP and SS conceived the project. IM, FP, SS, HP and SM collected the data. FP and SM conducted the data analysis with support from IM, CF, CK and HP. CF, CK and HP provided clinical input. All authors were involved in drafting the manuscript, and read and approved the final manuscript.
